# The War on Cancer: A Military Perspective

**DOI:** 10.3389/fonc.2014.00387

**Published:** 2015-01-26

**Authors:** Bryan Oronsky, Corey A. Carter, Vernon Mackie, Jan Scicinski, Arnold Oronsky, Neil Oronsky, Scott Caroen, Christopher Parker, Michelle Lybeck, Tony Reid

**Affiliations:** ^1^EpicentRx, Inc., Mountain View, CA, USA; ^2^Walter Reed National Military Medical Center, Bethesda, MD, USA; ^3^InterWest Partners, Menlo Park, CA, USA; ^4^CFLS LLC, San Jose, CA, USA; ^5^Moores Cancer Center, University of California San Diego, La Jolla, CA, USA

**Keywords:** RRx-001, military, epigenetic modulation, metronomic dosing, Optimox, war on cancer

## Abstract

Actually it has not quite happened yet, but almost imperceptibly, by degrees, we are learning to live with cancer. The “War on Cancer,” although generally successful in the pediatric population, has gradually been replaced with a kinder, gentler treatment paradigm that strives to contain and maintain with stalemate over checkmate, a strategy that may literally constitute the path to least resistance. The purpose of this review is (1) to critically examine the War on Cancer as a powerfully evocative metaphor that is directly responsible for a counterproductive and even potentially dangerous war-like cell-kill treatment paradigm, (2) to suggest that a reframing of this metaphor in less retaliatory and aggressive terms along with a shift in clinical practice from a maximalist to a minimalist strategy is more appropriate to the treatment of cancer, and (3) to draw on examples from the military sector as points of reference and comparison that closely parallel the three therapeutic “control and containment” strategies discussed in this review: (1) “Optimox-like” trial designs, (2) epigenetic modulation, and (3) metronomic dosing.

## Introduction

Actually it has not quite happened yet, but almost imperceptibly, by degrees, we are learning to live with cancer.

As improbable and counterintuitive as it sounds, the scorched earth, win-at-all-costs ethos from the “War on Cancer,” although generally successful in the pediatric population, has gradually been replaced with a kinder, gentler treatment paradigm that strives to contain and maintain with stalemate over checkmate, a strategy that may literally constitute the path to least resistance.

The live-and-let-live handwriting is already on the wall with strategies that include “Optimox-like” trial designs, metronomic dosing, and epigenetic modulation, discussed in this review; the reality, largely unacknowledged though it may be, is that the war on cancer may not be winnable in the majority of cases, as currently waged.

Chemotherapy and radiation are the ultimate stress test for cancer cells, leading to an unintended “survival of the fittest” response in which the most sensitive cells are culled from the treatment-resistant herd; inevitably the price of this selection pressure is the emergence of acquired resistance and therapeutic failure, making aggressive therapy a self-defeating process. Nature abhors a vacuum and fills it up with resistant tumor cells, which ultimately dooms the outcome to failure.

This is not to sound a pessimistic or defeatist note; to the contrary, it may be finally possible to imagine a world in the not-too-distant future when cancer is a chronic disease like HIV, COPD, or diabetes: if total eradication is impossible, except in certain circumstances, due to the development of acquired resistance, then one alternative is simply to hold the line: to box-in the tumor cells with a discrete, focused strategy of containment, a path between the extremes of all-out assault and appeasement, thereby turning metastatic cancer from a death sentence into a chronic disease.

The purpose of this review is (1) to critically examine the War on Cancer as a powerfully evocative metaphor that implies the promise of complete victory, motivates an overly strong desire to retaliate (“hit hard, hit fast, and hit often” in the words of Admiral Halsey) and is directly responsible for a counterproductive and even potentially dangerous war-like cell-kill treatment paradigm, (2) to suggest that a reframing of this metaphor in less retaliatory and aggressive terms along with a shift in clinical practice from a maximalist to a minimalist strategy is more appropriate to the treatment of cancer, and (3) to draw on examples from the military sector as points of reference and comparison that closely parallel the three therapeutic “control and containment” strategies discussed in this review: (1) “Optimox-like” trial designs, (2) epigenetic modulation, and (3) metronomic dosing. This review is warranted if only because the central tenet of the War on Cancer, tumor eradication, has remained essentially invariant for over 40 years despite the fact that, by and large, it has not succeeded.

In American strategic language, rollback is the doctrine that advocates regime change, by force if necessary (1). On the other end of the spectrum is containment, the application of judicious counterforce to prevent expansion. Proposed by a mid-level American diplomat, George F. Kennan, containment, which eschews rollback, was the cornerstone of the U.S.-Soviet policy to contain Russian expansive tendencies and prevent the cold war from turning hot (2).

Ever since Nixon’s War on Cancer declaration in 1971 (3) (though he never actually used that phrase), the majority of the medical profession has pursued the opposite approach and endorsed rollback not containment or the taking up of therapeutic arms against cancer to wipe it out in the same way that it has wiped out polio or smallpox. (Ironically, Nixon withdrew from Vietnam, only to have entered another war with cancer.) Since the war trope is clearly here to stay (witness the persistence of the War on Drugs, War on Poverty, War on Malaria, etc.), different military examples are cited herein to add perspective and context and to frame the discussion about the therapeutic examples highlighted in this review.

War brings with it a set of assumptions and these assumptions have dominated the discourse and infused the landscape of cancer treatment for over 40 years. The pharmaceutical industry talks in combative terms of weapons, targets, arsenals, armamentariums, therapeutic bull’s-eyes, silver bullets, and magic bullets; patients are “warriors” encouraged to “win the fight/crusade against cancer” and “conquer the disease” and oncologists, described as “cancer warriors or fighters,” attempt to achieve a maximal and rapid cell killing. Cancer drugs are still currently developed through this 40-year old system, to achieve the maximally tolerated dose (MTD), as if cancer were an enemy to be defeated through a massive or overwhelming display of force. Based on the fervor with which it is conducted, the War on Cancer seems closer to a hyper-aggressive military campaign than a treatment trope. Slowly, however, with the recognition that the War on Cancer is less a war than a quagmire, having, by and large, failed to a deliver on its promise of a decisive victory (4), less strident and less aggressive strategies have emerged.

In 2001, the publication of the seminal “Hallmarks of Cancer” by Drs. Hannahan and Weinberg (H&W), which refined the War on Cancer metaphor into a series of six unifying principles that control cell growth and metastasis and ushered in the era of targeted therapies challenged the medical community to take stock and recognize that the only way to defeat cancer was to better understand it. History’s timeless lesson from as far back as Sun Tzu, the Chinese general from the sixth century, is that the successful outcome of war critically depends on accurate intelligence and knowledge of the enemy. The H&W war doctrine describing the strengths and perhaps weaknesses of Mankind’s enemy (cancer) was updated and expanded in 2011 (5).

Quagmires, defined as impasses or deadlocks, have occurred repeatedly in the context of military and evolutionary conflicts where one side is a much more pronounced orchestrator of pressure. This pressure forces or induces the other side to evolve countermeasures to resist it. Thus, in Vietnam, Iraq, and Afghanistan (6), it was the U.S. that brought to bear overall superiority of manpower and firepower, creating a strong selection pressure for the enemy to evolve, adapt, and survive; this adaptation led to a vicious cycle of measures – countermeasures and a long, drawn-out war, in other words, a quagmire. In his book, “Learning to Eat Soup with a Knife,” a title based on the T. E. Lawrence (Lawrence of Arabia) observation about the conduct of guerilla warfare, Lieutenant Colonel John Nagl makes the case that the inability of the U.S. Army to adapt their tactics at the same rate as the Vietnamese, despite technological superiority, resulted in its failure to defeat the communist insurgency (7).

**Figure 1:**
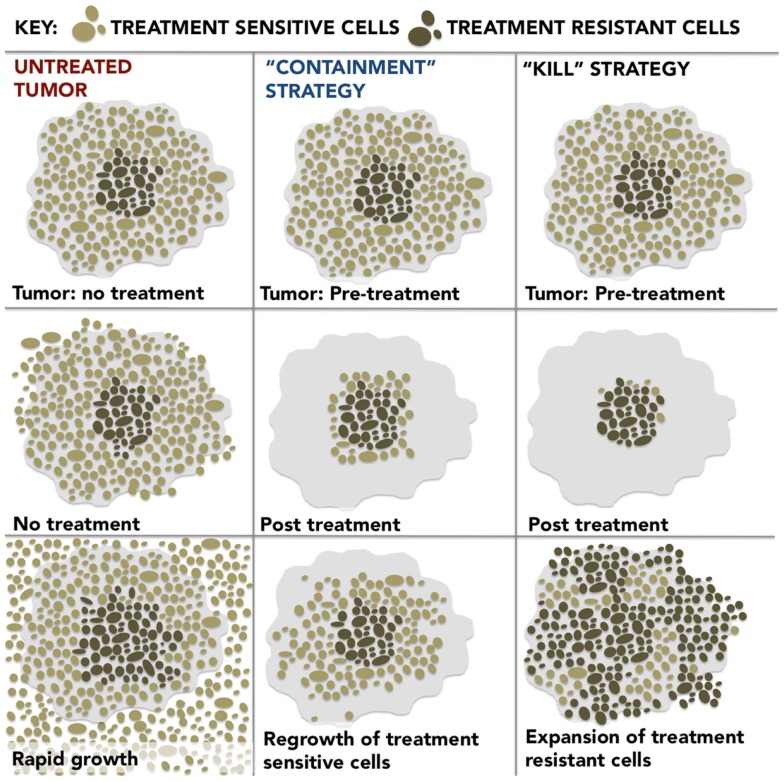
***Untreated Tumor*: the development of resistance is energetically expensive; therefore, the treatment-sensitive cells predominate in the untreated tumor and, by outcompeting the treatment-resistant cells for resources and space, inhibit their growth**. *“Containment” Strategy*: if treatment is optimized for stability rather than cure, a stable population of treatment-sensitive cells remain which suppresses the growth of resistant populations and results in prolonged patient survival. *“Kill” Strategy*: dose intensive treatment designed to kill a maximum number of cancer cells preferentially eliminates treatment-sensitive cells, actually promotes more rapid growth of the resistant population and leads to treatment failure and poor outcomes as a result.

Likewise, in the case of cancer, the lethal force of chemotherapy, designed to wipe out the tumor, may actually have the opposite effect: the chemosensitive cells, which normally keep the chemoresistant forms in check, by competing for scarce space and resources, are killed off. Cancerous tumors adapt in Darwinian fashion to their environment and evolve by clonal expansion and genetic diversification (8). As a result, the tumor emerges more treatment resistant than it was before (Figure 1).

Cancer is by no means monolithic; rather, it is a highly diverse and heterogeneous population of cells, with different degrees of overall fitness, even within a single tumor due to both genetic and environmental factors (9). In general, the most drug-susceptible cancer cells are located in the outer rim due to their proximity to vascularization. In contrast, like the Taliban and Al-Qaeda fighters in Afghanistan, who hide out and hole up in a complex warren of cave bunkers, the cancer cells in the inner regions of the tumor are able take refuge in hypoxic crevices and burrows created by an aberrant and dysfunctional tumor vasculature, safe from the predations of chemotherapy and radiation, which makes them phenotypically resistant. If the drug-sensitive clones on the periphery are preferentially eliminated with radiation and/or chemotherapy, then it is these resistant subpopulations that prevail and “hijack” the tumor.

According to the Red Queen hypothesis, a widely used concept from evolutionary biology, in reference to the character from the Lewis Carroll novella, “Through The Looking Glass,” who comments to Alice that “it takes all the running you can do, to keep in the same place,” co-evolution drives continuous adaptation and counter-adaptation just to stay even (10). Similarly, chemotherapy and radiation exert a strong selective pressure on the cancer cells to adapt often resulting in a no-win situation.

And so while the War on Cancer drags on officially without armistice or quarter unofficially signs of a tactical shift are already underway, with “Optimox-like” trial designs, epigenetic modulation, and metronomic dosing serving as the basis for discussion in this review.

## Optimox and “The Surge”

The Surge, as it was called, referred to a modest temporary increase in U.S. troops in 2007 in Iraq to quell an insurgency that was spiraling (surging) out of control, after which Bush’s plan was to return to pre-surge levels (11). An Obama-led troop surge, which took place in Afghanistan in 2010 with the limited objective to beat back the Taliban and Al-Qaeda and disrupt their momentum long enough for the Afghans to take over the fight (12), set the stage for the U.S. to withdraw from Afghanistan.

This surge strategy finds parallels in the “stop and go” Optimox 2 trial design (13) to manage oxaliplatin-induced neuropathy, where six cycles of FOLFOX induction were followed by infusional FU until tumor regrowth, at which point oxaliplatin was reintroduced, resulting in less neurotoxicity with comparable efficacy and survival.

Variations on this Optimox theme include

- a complete break in treatment after induction therapy until the tumor begins to regrow
- the modified “stop and go” of the Phase 3 Combined Oxaliplatin Neurotoxicity Prevention Trial (CONcePT) study in which patients were “on” oxaliplatin for a predefined interval of 4 months (FOLFOX and bevacizumab) and “off” oxaliplatin for a predefined interval 4 months (LV5FU2 plus bevacizumab). Despite early termination, CONcePT, which compared this intermittent oxaliplatin (IO) schedule to a conventional oxaliplatin (CO) “treat-to- failure” approach, demonstrated an impressive improvement in PFS from 7.3 months to 12.0 months with the stop-and-go strategy (14).

In a recent review of stop-and-go clinical trials from 2000 to 2013 entitled, “Continuous versus Intermittent Chemotherapy Strategies in Metastatic Colorectal Cancer: A Systematic Review and Meta-Analysis” by Berry et al. the authors concluded that compared to continuous chemotherapy, the intermittent strategies did not negatively impact on overall survival (OS) and “either maintained or improved quality of life (QOL)” (15).

An additional Optimox-like variation, called adaptive therapy, developed by Gatenby et al. (16) through mathematical modeling (a case of math versus malignancy), proposes treatment-for-stability in lieu of treatment-for-cure (17) to maximize survival. The premise is that the classic administration of maximum dose dense chemotherapy on a regular schedule favors the rapid growth of resistant subpopulations (18). Adaptive therapy advocates a Goldilocks approach, where the tumor burden is kept at a constant, stable level, not too much, not too little, but just right to maintain a sufficient number of chemotherapy-sensitive cells to inhibit the growth of resistant subpopulations. In this way, treatment is intermittent to coincide with spurts in tumor growth, and the emphasis is on containment not cure (19).

So pervasive and influential is the War on Cancer mindset especially in the U.S. that patients may refuse sporadic or intermittent therapy on the basis that it is preferable to hit the tumor hard and often – it may be for this reason that the Optimox trials were enrolled in Europe not U.S. – even though continuous treatment appears to result in pain without gain, i.e., increased toxicity to patients without improvement in overall survival, compared to a stop-and-go strategy.

## Epigenetic Modulation as a “Shaping Operation”

At times of war, the act of degrading and softening up an enemy in support of and in preparation for a decisive assault is called, in military parlance, a “shaping operation.” According to the Army Field Manual 3-0 (20), “*A shaping operation is an operation at any echelon that creates and preserves conditions for the success of the decisive operation. Shaping operations establish conditions for the decisive operation through effects on the enemy, population (including local leaders), and terrain… Shaping operations may occur throughout the operational area and involve any combination of forces and capabilities*.”

In many ways a “shaping operation” or, rather, a “*re*-shaping operation” describes the tumor remodeling action of the epigenetic agents, through beneficial re-expression of methylated and abnormally silenced genes to degrade and reverse resistance to subsequent therapy.

In a Phase 1/2 clinical trial of double epigenetic blockade with a combination of 5-azacitidine and entinostat in advanced, refractory non-small cell lung cancer (NSCLC), several patients demonstrated sustained and durable responses to their immediate next line of therapy *of 4 years or more* following progressive disease on azacitidine and entinostat (21, 22), leading the authors to hypothesize that the epigenetic therapy primed the cancer for response to subsequent treatment.

These observations are in agreement with another Phase 1 trial with the novel systemically non-toxic epigenetic agent, RRx-001, a double HDAC, and DMA methyltransferase inhibitor, where five colorectal cancer (CRC) patients were resensitized to previously failed FOLFIRI for 6 months or longer post-RRx-001 progression (23, 24), which suggests that RRx-001 epigenetically disrupts multiple cellular processes including chemoresistance, possibly through the de-repression of tumor suppressor genes like p53. Resensitization of chemorefractory tumors is a focus of several Phase 2 RRx-001 clinical trials in diverse cancers such as CRC, hepatocellular carcinoma (HCC), glioblastoma (GBM), NSCLC, small cell lung cancer (SCLC), malignant pleural mesothelioma, gastric and breast carcinomas.

In this way, epigenetic inhibition may represent a “kinder, gentler” approach in which the tumor is “reprogramed” during a preliminary “run-in” or priming period, similar to the shaping operation described in the Army Field Manual above, to respond to subsequent cytotoxic treatment, thereby increasing survival but not toxicity.

## Metronomic Dosing and Economic Sanctions

As an alternative to armed conflict and all-out war, economic sanctions have played an important role in U.S. foreign policy throughout the twentieth and twenty-first centuries as a strategy to isolate, destabilize, and cripple “bad actors,” countries with inimical agendas like Russia, Cuba, Iran, North Korea, Sudan, and Syria (25).

Metronomic chemotherapy involves regular, frequent systemic administration (26), i.e., without a rest period of cytotoxic agents such as cyclophosphamide and methotrexate at lower doses, to contain rather than kill the tumor via multiple cytostatic mechanisms (27), including the induction of senescence, immunostimulation, and antiangiogenesis, which drain energy and resources, similar to an embargo, without perpetrating a holocaust on beneficial chemosensitive cells. In a systematic literature analysis of low dose metronomic (LDM) chemotherapy, Lien et al. concluded that, despite the lack of definitive Phase 3 trial results and even the rarity of high quality Phase 2 studies, “LDM appears to be clinically beneficial and safe in a broad range of tumors” (28) including breast, colon, and gastric cancers. LDM is typically administered with twice daily weekday on-weekend off (5 days on, 2 days off) capecitabine (29, 30).

## Conclusion and Future Perspective

The phrase, The War on Cancer, evokes the liberation of enemy-held territory and regime-change operations, a kind of C-Day or “Operation Overkill.” The problem with this image is that it misrepresents cancer as an enemy that is defeatable when, in fact, in the case of cancer cells, victory is only achievable with total eradication, which may not be readily possible. The very act of trying to drive the cancer cells to extinction only succeeds for the most part in removing the moderates, i.e., the chemosensitive cells, and leaving behind the extremists, i.e., the chemoresistant cells, which makes the situation that much more desperate.

A middle course between appeasement and Armageddon is containment, which takes a long view, and eschews “rollback,” provided the tumor remains in its box. According to Gatenby et al. (18), drug-susceptible cancer cells predominate in an untreated environment, reflecting the extra energy and substrate costs of the resistance phenotype. While the repeated administration of MTDs may induce regression or remission, recurrence of even more malignant tumors usually follows. Akin to the dangers of bacterial resistance due to antibiotic overuse, an MTD dosing strategy may constitute overtreatment, which selects for rather than bypasses resistant phenotypes. In other words, without the selection pressure from chemotherapy and radiation, sensitive cells have the upper hand and keep the resistant cells at bay.

Despite this apparent lose–lose, Catch-22 scenario (damned if you treat, damned if you do not), a third strategy, containment, presents itself as a solution to keep the “long peace” by turning cancer into a clinically manageable chronic disease like diabetes or hypertension, which is controlled but not cured with medication. Hopefully a state of balance is reached, in which the net result of tumor growth and destruction is zero or close to zero, resulting in stabilization or non-progression of disease.

For this reason, a hard line strategy, which emphasizes cure over coexistence and an all-out war instead of a limited one, may only force cancer down its projected path to surpass heart disease as the leading cause of death by 2030 (31) in a kind of self-fulfilling prophecy. Instead of risking mutual annihilation, mutual survival demands that we contain and maintain the *status quo* and thereby turn a “hot” war into a cold one. Although it may chafe and go against the grain to play not to lose rather than to win, the strategies discussed herein suggests that with respect to cancer we have started to do just that, slowly.

## Conflict of Interest Statement

The authors declare that the drug RRx-001, mentioned in this review, is manufactured by EpicentRx, Inc., and the majority of the research and clinical trials associated with drug RRx-001 have been funded by EpicentRx, Inc.

## Abbreviations

CO, conventional oxaliplatin; CONcePT, Combined Oxaliplatin Neurotoxicity Prevention Trial; CRC, colorectal cancer; GBM, glioblastoma; HCC, hepatocellular carcinoma; H&W, Drs. Hannahan and Weinberg; IO, intermittent oxaliplatin; LDM, low dose metronomic; MTD, maximally tolerated dose; NSCLC, non-small cell lung cancer; OS, overall survival; QOL, quality of life; SCLC, small cell lung cancer.
